# Application of Mendelian randomization in the discovery of risk factors for coronary heart disease from 2009 to 2023: A bibliometric review

**DOI:** 10.1002/clc.24154

**Published:** 2023-09-19

**Authors:** Dayuan Zhong, Hui Cheng

**Affiliations:** ^1^ Nanhai Hospital of Traditional Chinese Medicine Jinan University Foshan China; ^2^ Guangdong Provincial Hospital of Integrated Traditional Chinese and Western Medicine Jinan University Guangzhou China

**Keywords:** bibliometric analysis, coronary heart disease, Mendelian randomization study, risk factors

## Abstract

Coronary heart disease (CHD) is a life‐threatening condition that poses a significant risk to individuals. Mendelian randomization (MR) is an emerging epidemiological research method that offers substantial advantages in identifying risk factors for diseases. Currently, there are ongoing CHD‐related MR studies. To gain comprehensive insights into the focal areas and trends of CHD‐related MR research, this study utilizes bibliometrics to conduct an in‐depth analysis of CHD‐related MR articles published in the core database of Web of Science (WOS) from 2009 to 2023. A search was performed to identify CHD‐related MR articles published between 2009 and 2023 in WOS. The data, including publication countries, research institutions, journals, citations, and keywords, were analyzed using the Bibliometrix R‐4.0 software package. A total of 111 articles published in 71 journals were included in the analysis. The journal with the highest impact factor (IF) was the *New England Journal of Medicine*. The articles were distributed across 24 categories within the 71 journals, with the highest number of publications falling under *Cardiac & Cardiovascular Systems*, *Medicine, General & Internal*, and *Genetics & Heredity*. Among the articles, 57 were published in Q1 journals, 42 in Q2 journals, 9 in Q3 journals, and 2 in Q4 journals. The most frequently published journals on CHD‐related MR were *Frontiers in Cardiovascular Medicine*, *Frontiers in Genetics*, and the *Journal of the American College of Cardiology*. A total of 963 authors participated in the 111 articles, with the majority affiliated with institutions in the United Kingdom, the US, and China. The national cooperation network revealed close collaborations between the UK and the US, as well as between the UK and China. The publication of the 111 articles involved 453 research institutions, with Oxford University, Bristol University, and Cambridge University being the most frequently involved institutions. Out of the 111 articles, only 62 were directly related to CHD and MR, with CHD being the outcome factor in 61 of them. These 61 articles investigated 47 exposure factors across eight categories. Among these factors, 10 had been studied in more than 2 articles. The findings concerning the impact of serum uric acid and omega‐6 fatty acids on CHD risk were not entirely consistent. Research in MR related to CHD has been gradually gaining recognition, with an increase in both its academic credibility and collaborative efforts within this field. Indeed, MR has facilitated the identification of risk factors associated with CHD. However, the relationship between these disease risk factors and CHD requires further investigation for clarification. Future MR studies on CHD could prioritize the elucidation and validation of contentious disease risk factors, thereby paving the way for a more comprehensive exploration of additional factors contributing to the onset of CHD.

## Graphical abstract

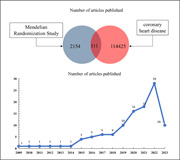

## 1 INTRODUCTION

Coronary heart disease (CHD) is the most common heart disease caused by atherosclerosis and poses a severe threat to individuals, often leading to fatal outcomes if left untreated. Global data indicate that CHD is one of the leading causes of death worldwide,^1^ and this holds true for China as well.^2^ Current treatment options for CHD primarily include drug thrombolysis and endovascular interventional surgery, which significantly improve the short‐term quality of life for CHD patients.^3^ However, these treatments also result in increased hospitalization costs for patients.^2^ The development of CHD is a gradual process stemming from progressive chronic inflammation, indicating that there is a window of opportunity to control the risk of CHD before it fully manifests.^4^ However, a specific and sensitive method for evaluating the risk of the disease during its early stages is still lacking.^3^ The “gold standard” for exploring the causal relationship between diseases is randomized controlled clinical trials (RCTs).^5^ Nevertheless, RCTs are often limited in the discovery of risk factors for CHD due to their high cost and time‐consuming nature.^5^ Mendelian randomization (MR) has emerged as an epidemiological research method with significant advantages in identifying risk factors for diseases.^6^ Unlike traditional confounding factors, MR analysis remains unaffected, thereby partially compensating for the limitations of RCTs.^5^, ^7^ MR is currently being widely employed in exploring the risk factors associated with CHD. However, the current status of MR research on CHD remains unclear. Bibliometric analysis (BA) is a technique for analyzing literature‐associated data, enabling a quick and intuitive understanding of the current status of MR studies on CHD.^8^, ^9^ Therefore, this study utilizes BA to systematically summarize the trends in MR research on CHD, institutional collaborations, and the existing research landscape and hotspots.

## 2 DATA AND METHODS

### 2.1 Literature search

The literature retrieval primarily focused on the core database of Web of Science (WOS), with a search conducted on May 15, 2023. The search terms used were “coronary heart disease” and “Mendelian Randomization Study.” The search scope was not limited, and the type of literature selected was within the WOS database. The specific formula used for literature search is as follows:

1. “Coronary heart disease” (all fields).
2. “Mendelian randomization study” (all fields)
3. #1 AND #2.

### 2.2 Statistical analysis

Once the relevant articles were retrieved, we exported the complete record citation data in bibtxt format. We carefully examined and categorized these articles based on their titles, abstracts, and full texts. To perform statistical analysis and visualization of articles related to CHD and MR, we utilized the bibliometrix^10^ package. This software is widely employed for literature analysis.^11^ Various analysis parameters were set based on the specific research content. By scrutinizing intricate relationships such as network structure, cross‐interactions, and dynamic changes within subject knowledge clusters, this tool assists researchers in uncovering the knowledge foundation, structure, and research frontiers within the respective field.

## 3 RESULTS

### 3.1 The results of literature retrieval and the trend of publication

**Figure 1:**
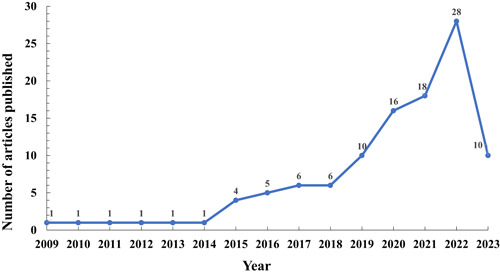
Annual publication of CHD‐related MR studies. CHD, coronary heart disease; MR, Mendelian randomization.

By conducting a comprehensive literature search, we identified a total of 111 articles related to CHD and MR. The earliest published CHD‐related MR study dates back to 2009. Initially, the number of MR studies focused on CHD remained steady at 1. However, from 2014 onwards, there has been a gradual increase in the number of studies, and the trend has accelerated significantly since 2018. Notably, in 2022, the number of CHD‐related MR studies peaked at 28, as depicted in Figure 1.

### 3.2 Articles published in journals

**Figure 2:**
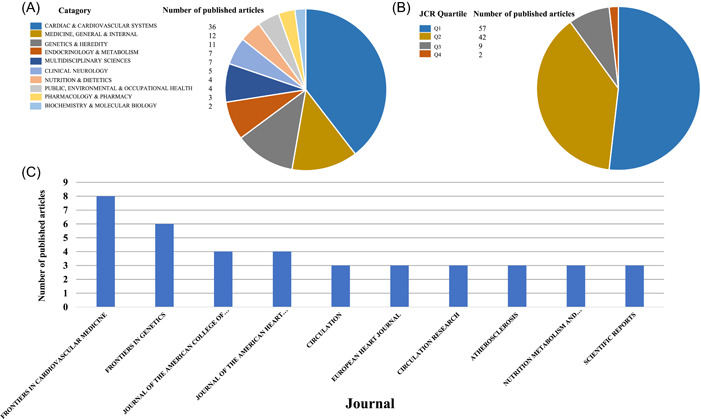
(A) Top 10 categories with the most articles published. (B) JCR quartiles of the articles. (C) Top 10 journals with the most articles published. JCR, journal citation reports.

Out of the 111 articles included in the analysis, 3 were published in journals indexed in the Emerging Sources Citation Index (ESCI), 1 in journals indexed in the Social Sciences Citation Index, and the remaining 107 in journals indexed in the Science Citation Index Expanded. These articles were published across a total of 71 journals. Among them, the *New England Journal of Medicine* had the highest IF of 176.082. Excluding the three ESCI journals, the lowest IF among the remaining 68 journals was found in *BMC Medical Genetics*, with an IF of 2.023. The articles covered a wide range of topics, falling into 24 categories across the 71 journals. The category with the highest number of published articles was *Cardiac & Cardiovascular Systems* (36 articles), followed by *Medicine, General & Internal* (12 articles), and *Genetics & Heredity* (11 articles), as illustrated in Figure 2A. One of the 71 journals did not have a Journal Citation Reports (JCR) quartiles (Q), and 1 article was published in that particular journal. Among the remaining 110 articles, 57 were published in journals classified as Q1 (top quartile), 42 in Q2, 9 in Q3, and 2 in Q4, as shown in Figure 2B. The most frequently published journals on CHD‐related MR were *Frontiers in Cardiovascular Medicine* (8 articles), *Frontiers in Genetics* (6 articles), *Journal of the American College of Cardiology* (4 articles), and *Journal of the American Heart Association* (4 articles), as depicted in Figure 2C.

### 3.3 Analysis of countries and institutions published in the article

**Figure 3:**
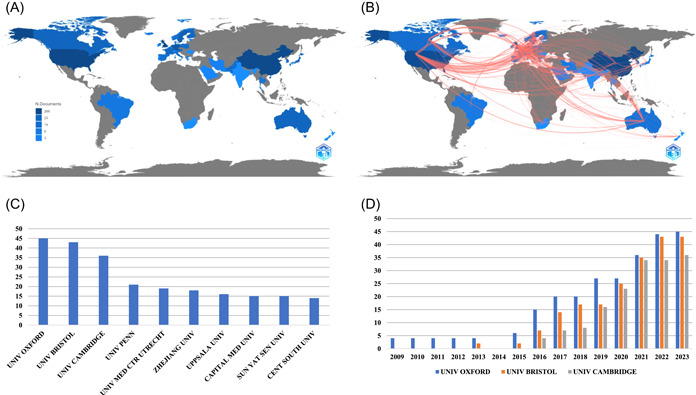
(A) Country distribution of authors. (B) Country cooperation. (C) Top 10 affiliated institutions with the most articles published. (D) Annual number of articles published by the top three affiliated institutions.

A total of 963 authors contributed to the 111 articles, with the majority of authors affiliated with institutions in the United Kingdom (266 authors), the United States (257 authors), and China (222 authors), as depicted in Figure 3A. The national cooperation network reveals close collaborations between the UK and the US, as well as between the UK and China, as shown in Figure 3B. A total of 453 affiliated institutions participated in the publication of these 111 articles. Among them, Oxford University had the highest level of involvement with 45 articles, followed by Bristol University with 43 articles, and Cambridge University with 36 articles, as shown in Figure 3C. Notably, Oxford University has been actively engaged in MR studies on CHD since 2009, while Bristol University and Cambridge University joined the field in 2013 and 2016, respectively. Over the years, the number of related publications has increased steadily, with Oxford University maintaining a leading position, as illustrated in Figure 3D.

### 3.4 Cooccurrence network analysis

**Figure 4:**
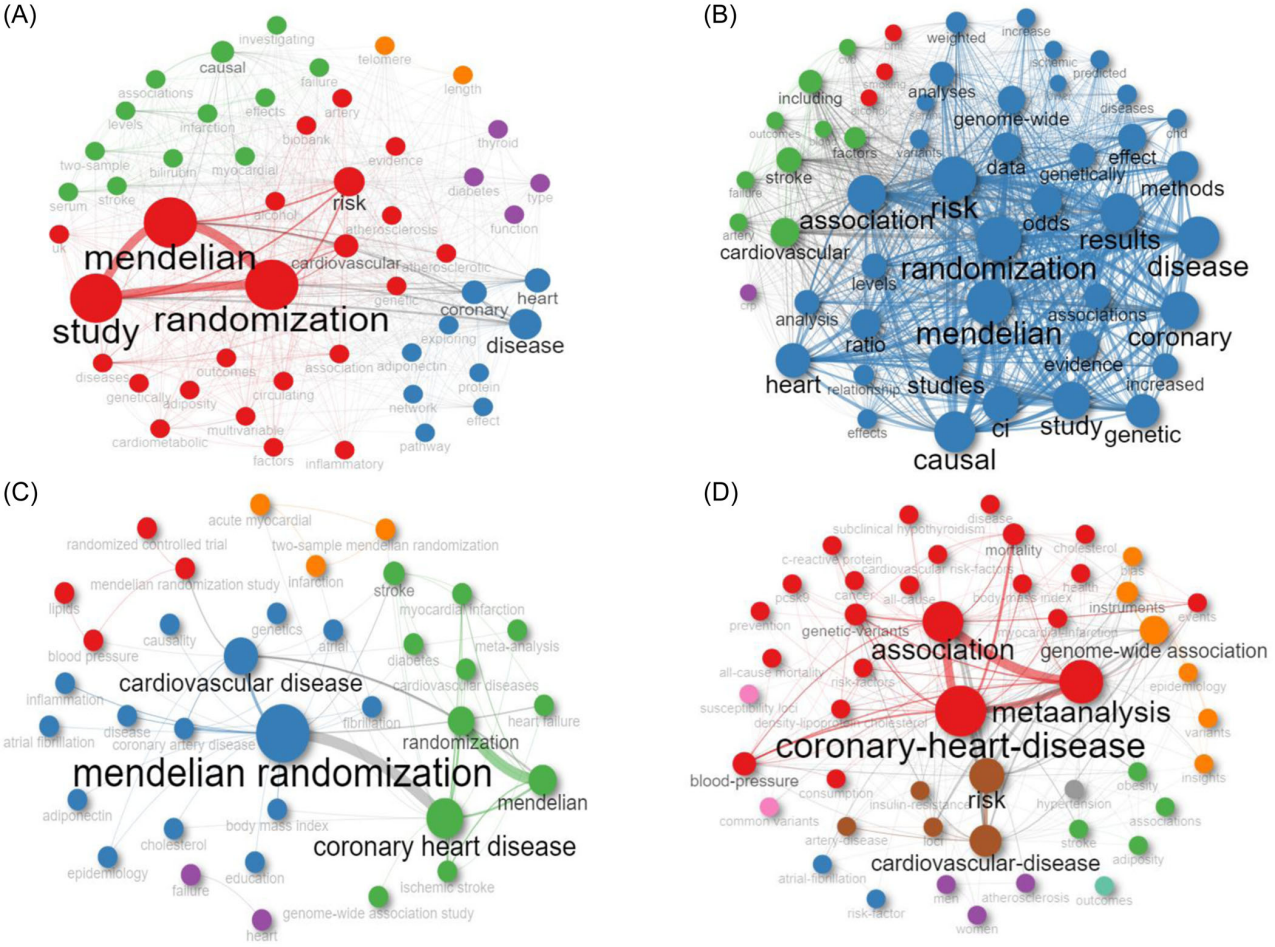
(A) Cooccurrence network of titles. (B) Cooccurrence network of the abstract. (C) The cooccurrence network of the author's keywords. (4) Cooccurrence network of keyword‐plus.

The cooccurrence network analysis of the title reveals that frequently appearing words are related to MR, such as Mendelian, randomization, and study, as depicted in Figure 4A. In the abstract, commonly used words revolve around MR, including Mendelian, randomization, and study. Additionally, there are CHD‐related terms like coronary, heart, and disease, as well as words indicating the study's objective, such as risk, causal, and association, as shown in Figure 4B. The author's keywords predominantly consist of MR and CHD‐related terms, such as MR, CHD, and cardiovascular disease, as illustrated in Figure 4C. Keyword‐plus words commonly include CHD‐related terms and words related to the research purpose, such as CHD, cardiovascular disease, risk, and association, as displayed in Figure 4D.

### 3.5 Analysis of the cited articles

In total, 111 publications have been cited a cumulative total of 3245 times, with an average of 29.23 citations per article. There are 10 publications that have received more than 100 citations and 17 publications that have received more than 50 citations. Notably, the most highly cited publication is the research paper authored by Elliott et al. published in 2009.^12^ Detailed information regarding the most highly cited articles is presented in Table 1.

**Table 1:** The 10 most cited articles.

Paper	Purpose	Conclusion	TC	TC/year
Elliott P (2009)^12^	To investigate whether CRP is associated with the risk of CHD.	There is no causal relationship between CRP and CHD.	480	32
Haycock PC (2017)^13^	To explore the causal relationship between telomere length and the risk of CHD.	There is a causal relationship between telomere length and the risk of CHD.	291	41.57
Ference BA (2015)^14^	To investigate the effect of NPC1L1 gene, HMGCR gene, or simultaneous polymorphism‐mediated natural random allocation on the risk of CHD by reducing LDL‐C.	The effect of lower LDL‐C on the risk of CHD mediated by polymorphisms in NPC1L1, HMGCR, or both is approximately the same per unit lower LDL‐C and log‐linearly proportional to the absolute exposure to lower LDL‐C.	237	26.33
Kleber ME (2015)^15^	To assess whether uric acid is an independent and causal cardiovascular risk factor.	Both uric acid and genetic risk scor were associated with cardiovascular death and sudden cardiac death.	176	19.56
Ference BA (2019)^16^	To assess whether the gene inhibition of ATP citrate lyase is associated with adverse outcomes, and whether it has the same effect as the gene inhibition of HMGCR for every 1 unit reduction in low‐density lipoprotein cholesterol levels.	Genetic variants that mimic the effects of ATP citrate lyase inhibitors and statins appear to reduce plasma LDL cholesterol levels through the same mechanism of action, and are associated with cardiovascular disease risk for each unit of LDL cholesterol reduction.	146	29.2
Lyall DM (2017)^17^	To explore the causal relationship between BMI and cardiovascular metabolic disease outcomes and pulse rate.	There is a causal relationship between elevated BMI and increased risk of cardiovascular metabolic diseases.	130	18.57
Hartwig FP (2017)^18^	To evaluate whether inflammatory biomarkers have an effect on the risk of developing schizophrenia.	There is a causal relationship between the protective effect of CRP and the increased risk of schizophrenia by sIL‐6R.	121	17.29
Keenan T (2016)^19^	To assess whether there is a causal relationship between serum uric acid levels and type 2 diabetes mellitus, CHD, ischemic stroke, and heart failure.	There is no causal relationship between circulating serum uric acid level and T2DM, CHD, ischemic stroke, or HF.	115	14.38
Prins BP (2016)^20^	To assess whether CRP has a causal relationship with immune, cardiac metabolism, and psychiatric disorders.	The evidence obtained cannot verify the causal effect of CRP levels on any other common physical and neuropsychiatric outcomes in this study.	111	13.88
Kamstrup PR (2012)^21^	To investigate whether lipoprotein (a) is a major risk factor for cardiovascular disease.	There is a causal relationship between Lpa and atherosclerotic stenosis.	111	9.25

Abbreviations: CHD, coronary heart disease; CRP, C‐reactive protein; TC, total citations.

### 3.6 Confirmed risk factors associated with CHD

Upon thorough examination of the 111 articles, we identified 7 reviews, 2 articles for which the full text was unavailable, and 40 articles that were either unrelated or not directly associated with CHD. Out of the total, only 62 articles were specifically focused on CHD and MR. Among these, CHD was considered as an outcome factor in 61 articles, while only 1 article treated CHD as an exposure factor.^22^ Notably, 2 of the 61 articles employed the reverse MR method.^23^, ^24^ Within the 61 articles, a comprehensive analysis of 47 exposure factors across 8 categories was conducted. Supporting Information: File 1 provides detailed information on these exposure factors, highlighting 10 factors that were the subject of more than 2 studies.

### 3.7 Interaction of results from multiple studies

Among the 47 exposure factors examined, there were 10 factors that received the most frequent attention in the studies. These factors include Type 2 diabetes, thyroid function, BMI, obesity, smoking, serum uric acid (SUA), omega‐6 fatty acids (O6FA), C‐reactive protein (CRP), LDL‐C, and telomere length. However, the conclusions regarding the impact of SUA and O6FA on the risk of CHD were not entirely consistent. While O6FA primarily yielded positive results, SUA produced both negative and positive findings, as indicated in Supporting Information: File 2.

## 4 DISCUSSION

A total of 111 articles related to CHD and MR were collected for this study. The earliest MR study on CHD was published in *JAMA* in 2009.^12^ This influential article investigated the causal relationship between CRP and CHD using MR research methods. Since its publication, it has garnered significant attention, receiving a total of 480 citations, averaging 32 citations per year. From 2009 to 2014, CHD‐related MR studies remained stable, but since 2014, there has been a gradual increase in research activity. This indicates the growing recognition and importance of MR research in the field of CHD. Journals in the JCR database are categorized and ranked into quartiles (Q) based on their IF within their respective disciplines. Q1 represents the top 25% of journals with the highest IF, while Q2, Q3, and Q4 represent subsequent quartiles. Q1 and Q2 journals tend to have higher citation frequencies and are generally considered influential in their fields. The findings of this study revealed that the majority of CHD‐related MR studies were published in Q1 journals (51.35%) and Q2 journals (37.84%), indicating the high recognition of MR studies in the field of CHD. Notably, some prestigious medical journals such as *NEJM, JAMA, and JACC* are among the Q1 journals, further emphasizing the impact of MR research on CHD.

A total of 111 articles involved 963 authors. The majority of authors hail from the United Kingdom, United States, and China, indicating a concentration of resources and research efforts in the field of CHD‐related MR studies within these countries. Notably, the University of Oxford, the University of Bristol, and the University of Cambridge have emerged as the leading institutions with the highest number of published CHD‐related MR studies. These renowned institutions have been actively involved in MR research, particularly Oxford University, which has been at the forefront of this field. The national cooperation network analysis reveals close collaborations between the UK and the US, as well as the UK and China. This can be attributed to the fact that many Chinese students choose to pursue their studies in the United Kingdom and the US, fostering research collaborations between these countries.^25^

The cooccurrence network analysis of the article titles and abstracts indicates that frequently appearing words are primarily MR‐related terms, such as Mendelian, randomization, and study. Additionally, CHD‐related terms like coronary, heart, and disease are also prominent. In terms of author‐provided keywords, MR and CHD‐related terms, such as MR, CHD, and cardiovascular disease, are frequently utilized. Similarly, the common keywords found in the keyword‐plus section include CHD‐related words and research‐purpose‐related terms. These findings provide strong evidence of the close association between the included literature and the fields of CHD or MR.

The article with the highest number of citations is a research paper published in 2009 by Elliott et al.^12^ This study aimed to investigate the association between plasma levels of CRP and the risk of CHD through a 19‐year clinical study spanning from 1989 to 2008. By analyzing genome‐wide association (GWAS) data of the population, the study concluded that there was no causal relationship between CRP and CHD.^12^ As the earliest CHD‐related MR study, the results of this article continue to guide the prevention and treatment of CHD. Moreover, it was published in the prestigious *Journal of the American Medical Association* (*JAMA*), which further boosted its influence and the number of citations. The second most cited article was a collaborative publication by the Telomeres Mendelian Randomization Collaboration.^13^ This study assessed the causal relationship between telomere length and the risk of 35 cancers and 48 non‐cancer diseases.^13^ As a MR study, this article provided extensive data covering multiple diseases and systematically analyzed their risks. This broad scope enhanced its influence across various disease disciplines. Furthermore, the article was published in *JAMA Oncology*, a journal established in 2014 that has gained significant prominence in the research community. The third most cited article was published by Ference et al.^14^ The objective of this study was to investigate whether the natural random allocation mediated by polymorphisms of the NPC1L1 gene, HMGCR gene, or both (targets of combined therapy) could reduce LDL‐C levels and subsequently lower the risk of CHD.^14^ The results indicated that the effects of lower LDL‐C levels mediated by NPC1L1, HMGCR, or both polymorphisms on CHD risk were similar. This article introduced the 2 × 2 factor Mendelian random research method. Similarly to the previous articles, it was published in the influential *Journal of The American College of Cardiology* (*JACC*), a leading journal in the field of heart disease. The top three most cited articles each year were published by Elliott et al., Telomeres Mendelian Randomization Collaboration, and Ference et al.^12^, ^13^, ^16^ Ference et al.'s article was published in the *New England Journal of Medicine* (*NEJM*), one of the most renowned journals in the field of global medicine. This article constructed a genetic score comprising independent genetic variations in genes encoding ATP citrate lyase (ACLY) and HMGCR, and explored the causal association between these scores and plasma lipid levels, lipoprotein levels, as well as cardiovascular events and cancer risk.^16^

In the 111 articles examined, a total of 47 risk factors associated with CHD were identified. These risk factors can be categorized into eight distinct factors: Disease, reproductive factors, society and policy, body constitution, habits and customs, blood index, drug, and genetic and gene‐related information. Among these risk factors, 10 have been the subject of repeated studies: Type 2 diabetes, thyroid function, BMI, obesity, smoking, SUA, O6FA, CRP, LDL‐C, and telomere length. However, the findings regarding the relationship between SUA and O6FA and the risk of CHD have shown inconsistencies. The impact of these two risk factors on CHD risk is still a matter of debate. Therefore, future research should focus on further investigating the strength of the causal relationship between these factors and CHD risk to establish more robust conclusions.

For instance, Keenan et al. directly investigated the association between SUA and CHD using 28 known SNPs related to SUA, obtaining results contradictory to those of Yang et al.^19^, ^23^ Efstathiadou et al. analyzed 28 SNPs and found differences only in the results of fixed‐effects IVW, while all other statistical methods yielded nonsignificant differences (*p* > .05).^26^ Kleber et al. also conducted MR analysis using 28 SNPs, but they narrowed down their analysis to 14 SNPs associated with multiple mediating risk factors through mediating MR, specifically examining the effects of these SNPs on CHD alone.^15^ Finally, the causal relationship between SUA and CHD has been established. Yang et al. conducted an analysis using 41 SNPs related to SUA and found a causal relationship between SUA and CHD (OR = 1.10; 95% CI: 1.06−1.15).^23^ However, it is worth noting that the sample sizes varied among the four studies included in the analysis. These differences in conclusions may be attributed to the number of SNPs included in the analysis and the sample size of the GWAS. Therefore, future MR studies should comprehensively analyze all GWAS data relevant to the exposure and outcome to draw more robust conclusions. Additionally, the mediating MR method should be employed to analyze the impact of different SNP‐associated mediating factors on the outcome, thus providing guidance for more reliable conclusions. Regarding O6FA and CHD, Liao et al. discovered a causal relationship through MR analysis.^27^ Zhuang et al. obtained consistent results using the network MR approach.^28^ Conversely, Mazidi et al. conducted a meta‐analysis on the association between O6FA and CHD and found no significant correlation.^29^ These discrepancies in the conclusions may stem from differences in research methods employed.

## 5 LIMITATIONS

First, our study solely relied on literature from the WOS database, potentially overlooking relevant articles published in other databases. Hence, there is a possibility of missing some pertinent literature on the research topic. Second, despite our rigorous search and screening process, a few unrelated articles might have been included, indicating the potential presence of additional articles that were not accounted for. Third, our analysis of CHD‐related MR studies was only preliminary and did not delve into a comprehensive examination. Consequently, the guidance provided for CHD‐related MR research is limited in its scope.

## 6 CONCLUSION

Despite the limitations of this study, it provides a comprehensive search and analysis of CHD‐related MR articles. The study successfully compiled published CHD‐related MR articles and examined various characteristics such as publication year, journal, country, institution, title, abstract, keywords, and highly cited articles. Furthermore, it summarizes the identified risk factors of CHD through MR methods and discusses their stability as well as the direction for future research. As a result, this study offers valuable insights and guidance for future CHD‐related MR research.

## AUTHOR CONTRIBUTIONS

*Determined the direction of the topic*: Dayuan Zhong. *Drafting of the manuscript, writing manuscript, literature search, data curation, and data analysis*: Dayuan Zhong and Hui Cheng. All the authors agreed to submit this paper.

## CONFLICT OF INTEREST STATEMENT

The authors declare no conflict of interest.

## Supporting information

Supporting information.

Supporting information.

## Data Availability

Requests for additional data may be granted upon reasonable request by contacting DY Zhong (dyzhong_medicine@126.com, +(86)13751728424).
