# Supplementary material for: Application of Mendelian randomization in the discovery of risk factors for coronary heart disease from 2009 to 2023: A bibliometric review
Source: Clin Cardiol. 2023 Sep 19;47(1):e24154. doi: 10.1002/clc.24154 (PMC10765999; doi:10.1002/clc.24154)
Supplement: Supplementary file 1 — Supporting information. [file CLC-47-e24154-s001.docx]

| **Supplementary file 1. Detailed information on exposure factors** | | |
| --- | --- | --- |
| **Categories** | **Exposure** | **Number of articles** |
| Disease | Type 2 diabetes | 4 |
|  | thyroid function | 2 |
|  | osteoarthritis | 1 |
|  | rheumatoid arthritis | 1 |
|  | atopic dermatitis | 1 |
|  | Insomnia and non vasomotor menopausal symptoms | 1 |
|  | Venous thrombosis, deep venous thrombosis and pulmonary embolism | 1 |
|  | stein-leventhal syndrome | 1 |
|  | COVID-19 | 1 |
|  | coronary artery calcification | 1 |
|  | asthma | 1 |
|  | inflammatory bowel disease | 1 |
| Reproductive factors | Age of first birth, number of live births, age of menarche and age of menopause | 1 |
|  | Age of first sexual intercourse and age of first birth | 1 |
|  | Age of natural menopause | 1 |
| Society and policy | education | 1 |
| Body constitution | BMI | 2 |
|  | obesity | 2 |
|  | waistline | 1 |
|  | height | 1 |
|  | body fat content | 1 |
| Habits and customs | smoking | 2 |
|  | alcohol | 1 |
|  | Excessive daytime siesta | 1 |
|  | physical activity | 1 |
| Blood index | serum uric acid | 4 |
|  | omega-6 fatty acids | 3 |
|  | CRP | 2 |
|  | LDL-C | 2 |
|  | plasma homocysteine | 1 |
|  | cytometry | 1 |
|  | Serum phosphate | 1 |
|  | High circulating cystatin C | 1 |
|  | High-sensitivity C-reactive protein | 1 |
|  | Circulating lipid mass spectrometry | 1 |
|  | adiponectin | 1 |
|  | matrix metalloproteinase | 1 |
|  | IL-6 and IL-6R | 1 |
|  | Vitamin E | 1 |
|  | Vitamin K | 1 |
|  | antioxidant | 1 |
| Drug | Interleukin 5 inhibitors | 1 |
|  | Prescription opioids | 1 |
| Genetic and gene-related information | Telomere length | 3 |
|  | Osteoprotegerin gene variation | 1 |
|  | Genetic scores of independent genetic variants in genes encoding ATP citrate lyase and HMGCR. | 1 |
