# Supplementary material for: Application of Mendelian randomization in the discovery of risk factors for coronary heart disease from 2009 to 2023: A bibliometric review
Source: Clin Cardiol. 2023 Sep 19;47(1):e24154. doi: 10.1002/clc.24154 (PMC10765999; doi:10.1002/clc.24154)
Supplement: Supplementary file 2 — Supporting information. [file CLC-47-e24154-s002.docx]

| **Supplementary file 2. Consistency of the conclusions of the 10 major exposure studies** | | | |
| --- | --- | --- | --- |
| Exposure | negative results | positive results | Consistency of conclusions |
| Type 2 diabetes | 0 | 4 | consistent |
| thyroid function | 2 | 0 | consistent |
| BMI | 0 | 2 | consistent |
| obesity | 0 | 2 | consistent |
| smoking | 0 | 2 | consistent |
| LDL-C | 0 | 2 | consistent |
| CRP | 2 | 0 | consistent |
| telomere length | 0 | 3 | consistent |
| serum uric acid | 2 | 2 | inconsistent |
| omega-6 fatty acids | 1 | 2 | inconsistent |
